# MMOSurv: meta-learning for few-shot survival analysis with multi-omics data

**DOI:** 10.1093/bioinformatics/btae684

**Published:** 2024-11-19

**Authors:** Gang Wen, Limin Li

**Affiliations:** School of Mathematics and Statistics, Xi’an Jiaotong University, Xi’an, Shaanxi 710049, China; School of Mathematics and Statistics, Xi’an Jiaotong University, Xi’an, Shaanxi 710049, China

## Abstract

**Motivation:**

High-throughput techniques have produced a large amount of high-dimensional multi-omics data, which makes it promising to predict patient survival outcomes more accurately. Recent work has showed the superiority of multi-omics data in survival analysis. However, it remains challenging to integrate multi-omics data to solve few-shot survival prediction problem, with only a few available training samples, especially for rare cancers.

**Results:**

In this work, we propose a *m*eta-learning framework for *m*ulti-*o*mics few-shot *surv*ival analysis, namely MMOSurv, which enables to learn an effective multi-omics survival prediction model from a very few training samples of a specific cancer type, with the meta-knowledge across tasks from relevant cancer types. By assuming a deep Cox survival model with multiple omics, MMOSurv first learns an adaptable parameter initialization for the multi-omics survival model from abundant data of relevant cancers, and then adapts the parameters quickly and efficiently for the target cancer task with a very few training samples. Our experiments on eleven cancer types in The Cancer Genome Atlas datasets show that, compared to single-omics meta-learning methods, MMOSurv can better utilize the meta-information of similarities and relationships between different omics data from relevant cancer datasets to improve survival prediction of the target cancer with a very few multi-omics training samples. Furthermore, MMOSurv achieves better prediction performance than other state-of-the-art strategies such as multitask learning and pretraining.

**Availability and implementation:**

MMOSurv is freely available at https://github.com/LiminLi-xjtu/MMOSurv

## 1 Introduction

Survival analysis of cancer patients has been a challenging hot topic for medical research field in the last few decades (Kourou *et al.* 2015, Nagy *et al.* 2021), which aims to predict the survival outcome from multiple explanatory variables. Although the American Joint Committee on Cancer has developed cancer staging references to quickly find important information about different types of cancers from the clinical data, such as tumor size and extent of tumor, high-throughput omics data such as gene expression have been popularly used to predict the survival outcome from the view of individual genomics. The major challenge in survival analysis is that a large amount of censored data may exist, for which the exact time point of death is unknown. This often happens when the study loses track of patients or the study is finished before observing the events of interest. Furthermore, although high-throughput omics data could help understand disease prognosis, their high dimensionality brings new difficulty for survival analysis, especially when the training samples are limited for rare cancers. Additionally, multi-omics data provide more insights for better understanding patients’ features, but they also introduce more challenges and opportunities for survival analysis when only a few training samples are available—a scenario referred to as few-shot learning (Wang *et al.* 2020a).

In survival analysis, the Cox proportional hazard (Cox-PH) model (Cox 1972) and the accelerated failure time (AFT) (Miller 1976) model are two kinds of widely used frameworks. The Cox-PH model assumes the proportional hazards and has become the most popular method in survival analysis. Recently, several approaches have been proposed to improve the Cox-PH model. For example, researchers have tried to combine regularization (Goeman 2010, Yang and Zou 2013) and multitask learning (Li *et al.* 2016a) with the Cox-PH model, which show good performance when available data are scarce. Compared with the Cox-PH model, the AFT model assumes a time-dependent hazard ratio, and quantifies the relationship between the log survival time and covariates by a regression model. The AFT model requires that the survival time of cancer patients follows a certain distribution, such as the Log-Normal or Weibull distribution (Lee and Wang 2003), making it less generic than the Cox-PH model. Besides the Cox-PH and the AFT models, many recent studies proposed to use machine learning methods for time-to-event data analysis. For example, Ishwaran *et al.* (2008) introduced random forests into survival prediction and proposed random survival forests, an ensemble tree algorithm that can handle right-censored data; and Khan and Zubek (2008) proposed to adopt the powerful support vector regression method for analysis of time-to-event data.

Deep learning, which has shown great success in a variety of tasks (Devlin *et al.* 2018, Zhang *et al.* 2020), has been widely used in survival analysis. Katzman *et al.* (2018) proposed DeepSurv method, which applies deep neural network techniques to the Cox regression and performs better than previous linear and nonlinear survival analysis models. High-throughput sequencing technologies have produced high-dimensional genomic data such as gene expression, which could be used for survival prediction and treatment improvement (Ching *et al.* 2018, Hao *et al.* 2019). For example, Cox-nnet (Ching *et al.* 2018) and CoxPASNet (Hao *et al.* 2019) used the multilayer networks to handle high-dimensional gene expression data for survival prediction. By computing the importance of genes, Cox-nnet could identify relevant genes and pathways for cancer prognosis. Compared with Cox-nnet, CoxPASNet adopts a sparse coding technique to reduce the overfitting caused by the high dimensionality and low-sample size of the data, and shows significantly better performance. Similarly, Kim *et al.* (2020) put forward a VAECox algorithm involving two-stage parameter optimization process, which first learns a VAE model on RNAseq data from multiple cancer datasets, and then initializes the parameters of the survival prediction model for a specific cancer with the learned weights.

For specific rare cancers, such as mesothelioma (MESO), which occurs in the thin layer of tissue covering the majority of the internal organs, and uterine carcinosarcoma (UCS), which starts in the outer layer of the uterine muscle, the incidence rates are 5 per million and 7.5 per million, respectively. The extremely low incidence rate means that, in practice, only a few training samples are available for learning. Deep learning methods, when applied to a few data samples with high-dimensional features, are prone to overfitting. One strategy is to leverage the abundant data from the other common relevant cancers to improve the survival prediction of the rare cancer. Typically, transfer learning (Pratt 1992) and meta-learning (Finn *et al.* 2017, Amit and Meir 2018) approaches are practicable alternatives to solve this problem. Transfer learning (Li *et al.* 2016b) first trains a model for relevant tasks with abundant samples and then fine-tunes the model for the target task. Another strategy, namely meta-learning, aims to address the “learning to learn” problem and has gained much attention in recent years. The basic idea of meta-learning is to train a meta-model by integrating prior knowledge from a variety of relevant training tasks, such that the meta-model is capable of learning a new task with very few data and training iterations. Qiu *et al.* (2020) introduced meta-learning strategy into few-shot survival analysis with gene expression data, which transfers knowledge from relevant cancers with large amounts of training data to the target cancer, to improve survival prediction in few-shot setting. The experimental results show that, compared to transfer-learning, meta-learning is a remarkably more effective approach of knowledge transfer when there are very limited training samples for the specific target cancer.

Multi-omics learning methods (Huang *et al.* 2019, Wen and Li 2023), which can help understand inter-patient discrepancy in molecular data by exploring relationships among different omics data, have been shown to be more promising than single-omics methods for survival prediction. For example, HFBSurv method (Li *et al.* 2022), which explores complex relations between intra-omics and inter-omics by utilizing factorized bilinear models, can deeply mine diverse omics-specific and cross-omics information among different omics data to remarkably improve survival prediction. Different from HFBSurv method that takes feature fusion step by step, GCGCN method (Wang *et al.* 2020b) integrates multiple sample similarity matrices from different omics using the SNF algorithm (Wang *et al.* 2014) to capture complementary neighborhood information among multi-omics data. Similarly, high-dimensional features make it challenging to integrate multi-omics data. To address the limitation, meta-learning was introduced for multi-omics survival analysis to improve the performance of survival prediction (Cho *et al.* 2023). Although meta-learning methods have shown certain superiority in multi-omics survival prediction, it remains challenging to determine how best to utilize meta-learning methods to solve few-shot problem in multi-omics survival analysis.

In this work, to solve the few-shot problem of multi-omics survival analysis, we propose the meta-learning framework for multi-omics few-shot survival analysis (MMOSurv) approach which learns the meta-knowledge extracted across tasks from relevant cancer multi-omics datasets, to quickly recognize discriminative patterns for survival prediction using a few training samples from the target cancer task. Given that exploring multi-omics relations by representation learning may be possible to overcome the paucity of training data to some extent, we proposed to introduce meta-learning into multi-omics survival analysis method with representation learning, which could improve prognosis prediction for multi-omics few-shot task on target cancer by learning meta-knowledge of cross-correlations among different omics. Specifically, MMOSurv first learns a suitable initialization of parameters for the multi-omics survival model from multi-omics data of relevant cancers, and then adapts the parameters quickly and efficiently for the target cancer task with a few training samples. To verify the effectiveness of our MMOSurv approach on few-shot problem of multi-omics survival analysis, we conducted comprehensive experiments on real-world cancer datasets. The results show that MMOSurv can alleviate the omics-bias problem by exploring relationships and similarities among different omics data and achieve better performance than single-omics meta-learning methods in few-shot problem. Furthermore, MMOSurv has been shown to perform significantly better for multi-omics few-shot survival prediction when leveraging high-dimensional multi-omics data from relevant cancers, compared with other state-of-the-art strategies such as multitask learning and pretraining.

## 2 Materials and methods

Multi-omics survival data consist of three components: multi-omics features $\left\{X^{\left(1\right)},\ldots ,X^{\left(v\right)}\right\}$ such as gene expression data and microRNA expression data, censoring indicator δ and observed time *O*, where *v* represents the number of omics. Generally, we denote the true survival time and the censoring time of patients by *T* and *C*, respectively, and assume that they are independent of each other. When the event of death happens before censoring (δ=1), the observed time *O* is the true survival time *T*; otherwise when δ=0, *O* is equal to the censoring time *C*.

The primary goal of multi-omics survival analysis is accurate survival prediction by deeply exploring and mining relations among different omics in an effective way. Though existing multi-omics survival methods can achieve excellent performance by integrating information from different sources, these methods do not work effectively when there are only a very few training samples and the dimensionality of features is very high. To solve few-shot problem of multi-omics survival analysis, we propose MMOSurv method which learns the meta-knowledge extracted across tasks from relevant cancer multi-omics datasets, to quickly recognize patient-distinguishing pattern for survival prediction with a few training samples from target cancer task. In this section, we first introduce the multi-omics survival analysis method based on representation learning, and then explain how to use meta-learning to optimize parameters of the multi-omics survival model.

### 2.1 Multi-omics survival prediction model

**Figure 1.**
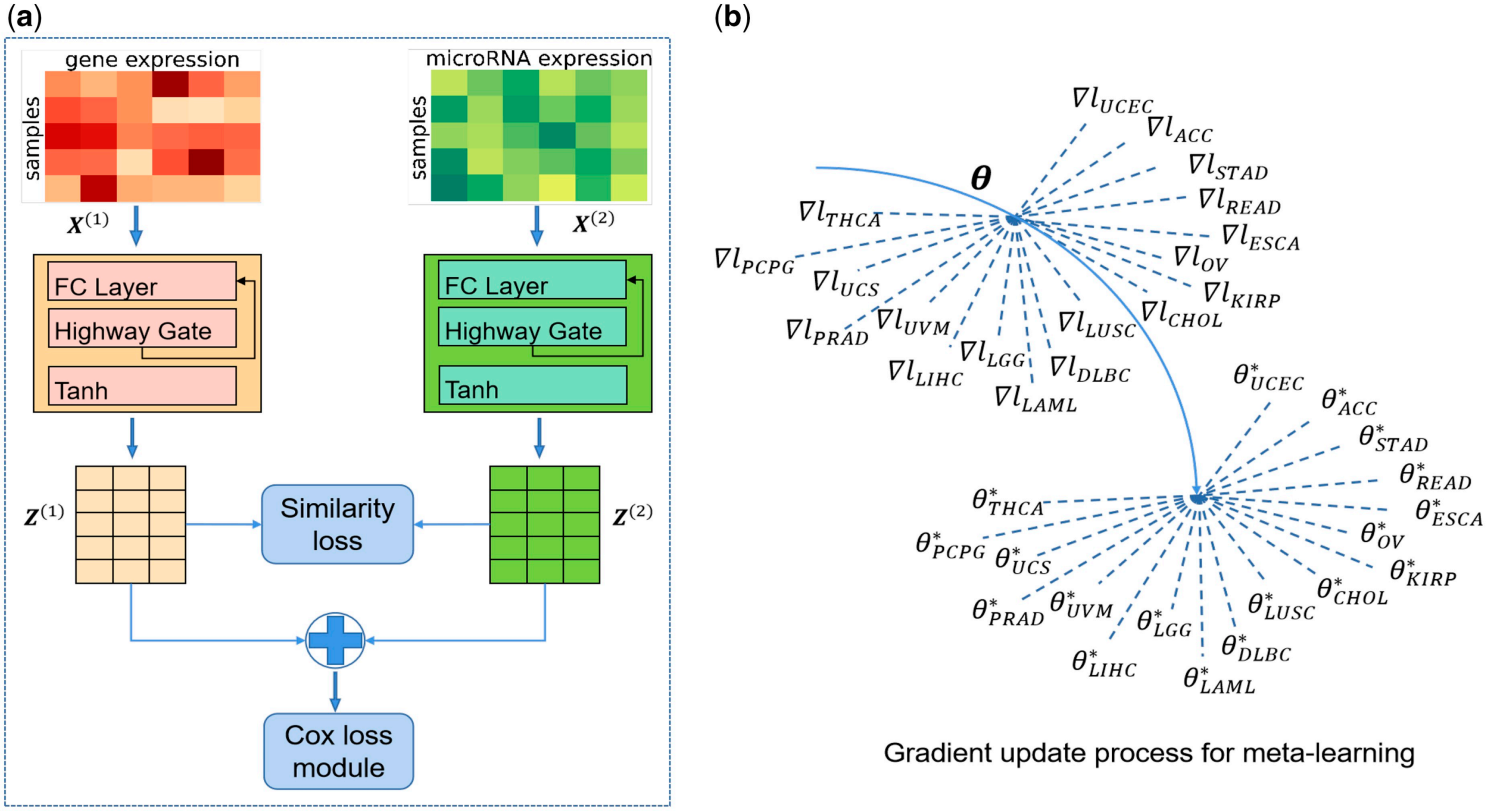
(a) Structure of multi-omics survival prediction model: representation learning projects different omics data in the same feature space by the similarity loss. Combining Cox hazard loss with the similarity loss, the algorithm can learn the similar discriminative feature representation of multi-omics data for survival prediction. (b) Meta-learning learns a suitable parameter initialization θ from tasks drawn from multi-omics data of relevant cancers that can be fine-tuned quickly on target cancer task.

In this work, we first construct a multi-omics survival prediction model by exploring the relationships between gene expression data and microRNA expression data. Our multi-omics survival prediction model is presented in Fig. 1a. For high-dimensional gene expression data $X^{\left(1\right)}=[x_{1}^{\left(1\right)},\ldots ,x_{n}^{\left(1\right)}]$ and microRNA expression data $X^{\left(2\right)}=[x_{1}^{\left(2\right)},\ldots ,x_{n}^{\left(2\right)}]$, we used two 5-layer highway networks (Srivastava *et al.* 2015) to map $X^{\left(1\right)}$ and $X^{\left(2\right)}$ into same embedding spaces and obtained the corresponding 80-dimensional feature representations $Z^{\left(1\right)}=[z_{1}^{1},\ldots ,z_{n}^{1}]$, $Z^{\left(2\right)}=[z_{1}^{2},\ldots ,z_{n}^{2}]$. The highway network, which is inspired by LSTM recurrent neural networks, makes use of a learned gating mechanism to regulate gradient flow between neural network layers, as shown below:

$$x_{\text{out}}=H(x_{\text{in}})*T(x_{\text{in}})+G(x_{\text{in}})*(1-T(x_{\text{in}})),$$

where *G* and *H* represent linear and nonlinear mappings, respectively, and *T* represents the transformation gate.

Previous research has confirmed the existence of remarkable cross-correlations among different data types (Gevaert *et al.* 2013) and exploring these relations by representation learning, to a certain extent, can overcome the paucity of training data and greatly improve the prognosis prediction process. Inspired by prior work (Cheerla and Gevaert 2019), we assume that the similarity between the feature representations from different omics for the same patient is maximized, whereas that for different patients is minimized. Specifically, deep unsupervised representation learning defines the similarity loss $l_{\text{sim}}(\varphi )$ as follows:

(1) $$\begin{array}{c} l_{\text{sim}}(\varphi )=\max(0,M-({\text{sim}}_{\varphi }(z_{i}^{1},z_{i}^{2})-{\text{sim}}_{\varphi }(z_{i}^{1},z_{j}^{2}))),\\ {\text{sim}}_{\varphi }(z_{i}^{1},z_{i}^{2})=\frac{{\left(z_{i}^{1}\right)}^{T}*z_{i}^{2}}{\left||z_{i}^{1}||||z_{i}^{2}|\right|}, \end{array}$$

where φ represents the parameters of highway network. Note that we use margin-based, hinge-loss formulation to ensure stability of algorithm, such that when we optimize the loss function, we only focus on the situation that the similarity between different-object feature representations falls within a margin *M* of the similarity between same-patient feature representations. As a result, different omics of the same patient have as similar feature representations as possible, while the feature representations of different patients are as far apart as possible. As mentioned above, we can recognize important and meaningful patterns that distinguish patients by representation learning.

More importantly, the survival prediction model should predict the survival outcome for the patients accurately based on the 80-dimensional feature representations $\left\{z_{i}^{1},z_{i}^{2}\right\}$. The traditional Cox-PH model (Cox 1972), which assumes that hazard function has a multiplicative form $\lambda (t|x_{i})=\lambda_{0}(t)\;\text{exp}(\beta^{T}x_{i})$ and adopts the partial likelihood to estimate the model parameters, is widely used in the survival analysis. By combining Cox-PH model with multi-omics representation learning, the total loss of our multi-omics survival prediction model is

(2) $$\begin{array}{c} l(\varphi ,\beta )=l_{\text{cox}}(\beta )+l_{\text{sim}}(\varphi ),\\ l_{\text{cox}}(\beta )=-\frac{1}{n}\sum_{i=1}^{n}\delta_{i}(h_{\beta }(z_{i})-\text{log}\;\sum_{j:O_{j}>O_{i}}e\text{xp}(h_{\beta }(z_{j}))), \end{array}$$

where $z_{i}=(z_{i}^{1}+z_{i}^{2})/2$, and $h_{\beta }$ represents the neural network model to perform survival prediction. Based on overall loss, the multi-omics survival prediction model attempts to learn similar discriminative feature representation from different omics for survival prediction.

### 2.2 Meta-learning for few-shot survival analysis

With only a few samples, it is difficult to learn the parameters θ={φ,β} in the multi-omics survival prediction model. To deal with the few-shot problem of multi-omics survival analysis, we introduce meta-learning strategy to optimize the parameters θ of the model. Meta-learning assumes that there is a task distribution p(T) and aims to learn quickly and efficiently for a previously unseen task sampled from this distribution. The process of training the parameters θ based on meta-learning is composed of two phases: a meta-learning phase, and a final learning phase. The starting point of the meta-learning phase is to learn the model parameters that are sensitive to loss functions of a variety of different tasks drawn from the distribution p(T). Specifically, the meta-learning phase learns suitable initialization parameters for the model, and when simply fine-tuning the parameters, the model can produce maximally effective behavior on any learning task from p(T). It makes sure that during final learning phase the model can generalize well on a new task with a few training samples and training iterations (Finn *et al.* 2017, Nichol *et al.* 2018), without overfitting.

Specifically, we learn an initialization for the parameters θ from multi-omics data of relevant cancers, as illustrated in Fig. 1b, such that when we optimize these parameters θ on target cancer task, learning is fast—i.e. the model generalizes well with a very few training samples from the target cancer task. MAML (Finn *et al.* 2017) and Reptile (Nichol *et al.* 2018) are two common meta-learning methods. Compared with MAML, Reptile does not require splitting the training set and the test set for each learning task in meta-learning phase, and is easier to understand and implement. In this work, we adopted the first-order Reptile method to learn a suitable initialization of parameters for multi-omics survival model. The meta-learning phase of Reptile consists of two parts: inner-loop and meta-loop. The inner-loop learns task $T_{\tau }$ drawn from the training samples of relevant cancers, given initialization parameters θ, as shown below:

$$\begin{array}{c} \theta_{\tau }^{0}=\theta \\ \theta_{\tau }^{1}\leftarrow \theta_{\tau }^{0}-\alpha \nabla l(\theta_{\tau }^{0})\\ \cdots \\ \theta_{\tau }^{k}\leftarrow \theta_{\tau }^{k-1}-\alpha \nabla l(\theta_{\tau }^{k-1}) \end{array}$$

where $\theta_{\tau }^{k}$ is the parameters learned by the model taking *k* iterations on task $T_{\tau }$, $l(\theta_{\tau }^{k-1})$ is the loss of task $T_{\tau }$ calculated on the parameters $\theta_{\tau }^{k-1}$, the symbol ∇ represents differential operation, and α is the learning rate of inner-loop. Note that the inner-loop learns *m* tasks simultaneously, and the learning process of each task has the same initialization parameters θ and is done separately. Next, the meta-loop defines $\frac{1}{m}\sum_{\tau =1}^{m}\left(\theta_{\tau }^{k}-\theta \right)$ as a gradient to update the parameters θ, as followed:

(3) $$\theta \leftarrow \theta +\gamma \frac{1}{m}\sum_{\tau =1}^{m}\left(\theta_{\tau }^{k}-\theta \right),$$

where γ is the learning rate of the meta-loop optimization algorithm. In fact, meta-loop regards *m* tasks as *m* learning samples when updating the parameters. We repeated the above process of parameter update until reaching the maximum number of iterations.

In the final learning phase, we first initialize the model with the meta-learnt parameters θ, and then fine-tune the model with a few training samples from target cancer task.

## 3 Results

### 3.1 Datasets and preprocessing

The Cancer Genome Atlas (TCGA) (Zhu *et al.* 2014) provides multi-omics data for more than 11 000 samples spanning 33 cancer types. In our study, we downloaded RNASeq data for gene expression and miRNA-Seq data for microRNA expression to evaluate the MMOSurv method. First, we filtered out the genes/microRNAs with NA values exceeding 10%, and adopted median strategy to impute missing feature values. Second, we performed logarithm transform over the data and removed noise-sensitive features whose values remain almost unchanged across samples. Then we normalized the log-transformed data to ensure each feature exhibits zero mean and unit variance. In addition, many patients do not have all data available and we only kept the patients with matched gene expression and microRNA expression data. Finally, the data contain 9771 samples with 17 790 gene features and 1881 microRNA features from 32 cancer types.

We selected nine common cancer types as target cancers, which are of clinical interest and have more than 300 [except Esophageal Carcinoma (ESCA)] training samples for different benchmarking training schemes. The nine cancer types selected from TCGA are listed below: Kidney Renal Clear Cell Carcinoma (KIRC), Breast Invasive Carcinoma (BRCA), Colon Adenocarcinoma (COAD), Lung Adenocarcinoma (LUAD), ESCA, Uterine Corpus Endometrial Carcinoma (UCEC), Liver Hepatocellular Carcinoma (LIHC), Cervical squamous cell carcinoma and endocervical adenocar-cinoma (CESC) and Bladder Urothelial Carcinoma (BLCA). To further validate the performance of MMOSurv approach on the few-shot problem of multi-omics survival analysis, we selected two additional rare cancer cohorts: MESO and Uterine Carcinosarcoma. Table 1 presents a summary of the 11 target cancer datasets.

**Table 1.** Summary of the target cancer datasets.

Dataset	Patients	Prop.Censored	Dataset	Patients	Prop.Censored
BRCA	1021	0.861	CESC	304	0.763
KIRC	508	0.671	COAD	439	0.770
UCEC	533	0.835	LIHC	366	0.650
LUAD	496	0.637	BLCA	402	0.562
ESCA	164	0.610			
UCS	56	0.393	MESO	84	0.143

### 3.2 Experimental setting

**Figure 2.**
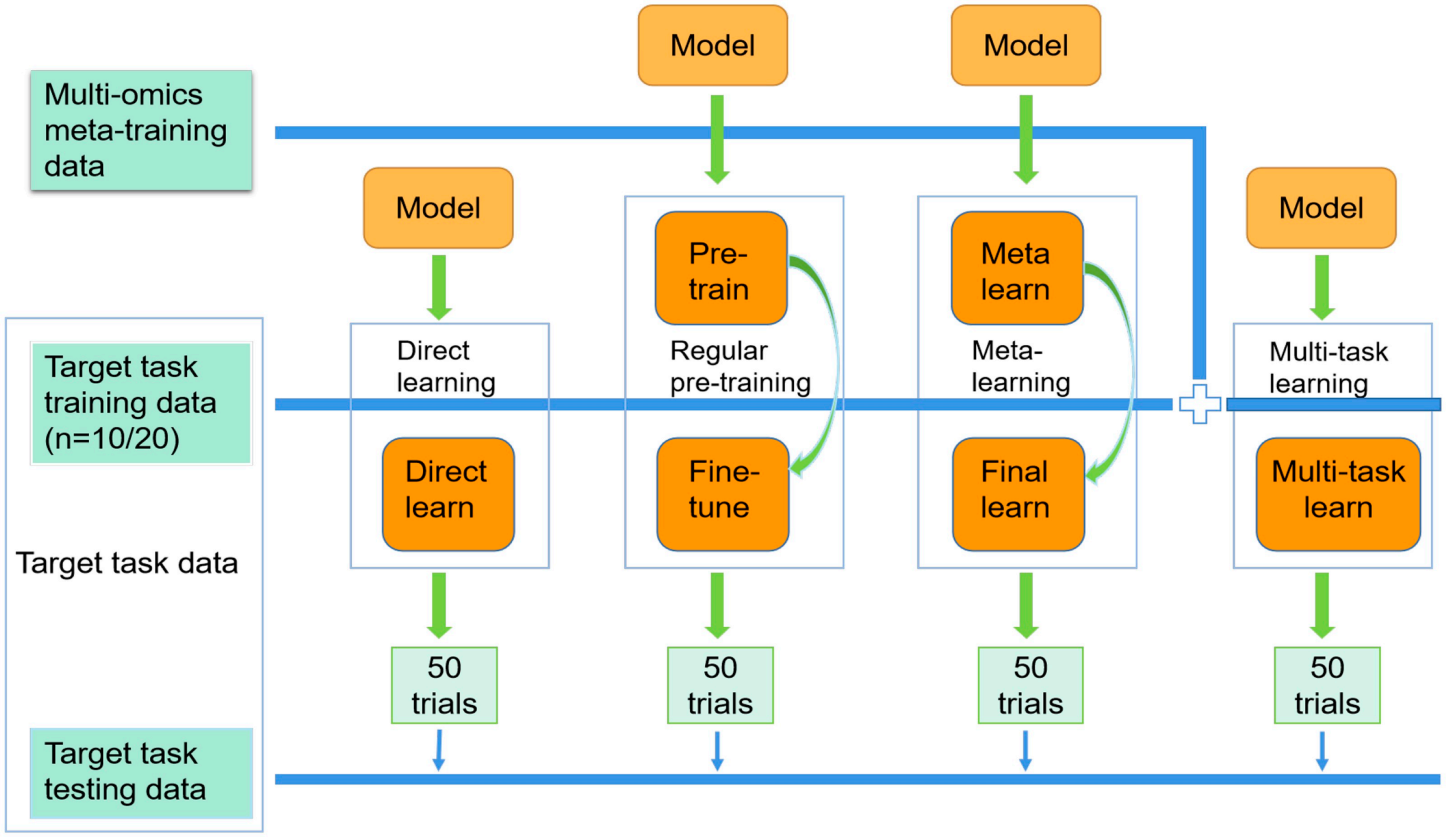
Data flow schematic. We compared meta-learning, pretraining, multitask learning and direct learning methods based on multi-omics data in few-shot (10/20) setting.

In order to assess the performance of our method MMOSurv in multi-omics few-shot survival analysis problem, we compared it with single-omics meta-learning methods, multi-omics meta-learning method without similarity constraint (WO_similarity), and several alternative multi-omics training schemes (Fig. 2): direct learning, regular pretraining and multitask learning.

- Meta-learning on single-omics initially learns meta-knowledge across tasks from meta-training dataset with single-omics, and then learns task-specific information from the target task with a very few training samples.
- WO_similarity, compared to MMOSurv, removes the similarity constraint of survival prediction model and does not explore the cross-correlations among different omics in the process of meta-learning.
- Direct learning only uses the very few available training samples from the target task to train survival prediction model.
- Pretraining (Xie and Richmond 2018) first trains survival prediction model on relevant cancer datasets and then fine-tunes on the target cancer task, without explicitly focusing on learning a parameter initialization that one or more gradient steps can achieve large improvement on new tasks.
- Multitask learning (Zhang and Yang 2022) simultaneously learns the auxiliary task on relevant cancer datasets and the target task from specific cancer training dataset. Multitask learning utilizes information from the relevant task to influence the learning of the target task through parameter sharing, making the target task perform better.

In our experiments, we collected multi-omics data of relevant cancers from TCGA as meta-training dataset. We randomly extracted a few samples stratified by censoring status, with 25% uncensored samples and 75% censored samples, from the meta-training dataset as a survival prediction task, and constructed a variety of different tasks to learn. The target cancer dataset (independent with the meta-training dataset) is split into training data and test data, stratified by censoring status. For the nine common target cancers, 20% of the data are used for testing; and for the two rare target cancers, 50% of the data are used for testing. To evaluate the performance of multi-omics meta-learning method MMOSurv in few-shot setting, we randomly drew 10/20 samples stratified by censoring status from the training dataset of target cancer as its training data. The small sample size of 10/20 was used for the target task since it is usual in studying rare diseases (Qiu *et al.* 2020). To comprehensively evaluate MMOSurv method, we randomly sampled the target task with 10/20 training samples 50 times. Finally, to ensure the robustness of our results, we repeated the above process 20 times and reported the mean of the results for each method. It is of note that, in this study, we evaluated the performance of MMOSurv approach in multi-omics few-shot survival prediction with two metrics: *C*-index and AUC (Chen *et al.* 2012). We provide detailed definitions of the *C*-index and AUC metrics in Section 12 of the Supplementary Materials.

To avoid overfitting, for all methods, we only searched for hyper-parameters in the 20-shot scenario and applied the chosen parameters to 10-shot scenario. For meta-learning method, in the inner loop, we set optimization algorithm to batch gradient descent and determined learning rate from [1e-1, 5e-2, 1e-2, 5e-3, 1e-3], tested batch size of 50, 100, 200, 400, and 800 for task $T_{\tau }$, searched the number *k* of gradient iteration steps from [5, 10, 20]; in the meta loop, we set optimization algorithm to Adam, searched learning rate from [5e-3, 1e-3, 5e-4, 1e-4, 5e-5], and chose the number *m* of sampling tasks from [3, 5, 10]. For multitask learning and regular pretraining, we determined learning rates with grid search on a grid of [5e-3, 1e-3, 5e-4, 1e-4, 5e-5] for Adam and tested batch sizes of 50, 100, 200, 400, and 800 for learning tasks extracted from relevant cancer datasets.

### 3.3 Performance evaluation of MMOSurv

In the following, we evaluated the performance of MMOSurv for multi-omics few-shot survival analysis on the nine common target cancer datasets in terms of *C*-index and AUC. Table 2 and Supplementary Table S1 report the mean values of the *C*-index and AUC obtained by different methods on each dataset, respectively. On one hand, compared with single-omics meta-learning methods, our multi-omics meta-learning method MMOSurv could achieve higher *C*-index and AUC values than single-omics methods. This indicates that MMOSurv can effectively take advantage of meta-information of similarities and relationships between different omics data from relevant cancer datasets, to quickly learn patient-distinguishable pattern for survival prediction with only a few training samples from the target cancer.

**Table 2.** C-index of different approaches in few-shot (10/20) setting.

	Data	Method	BRCA	KIRC	BLCA	CESC	LIHC	COAD	LUAD	UCEC	ESCA	Avg.ranking
10-shot	Gene	Meta-learning	0.639	0.672	0.632	0.682	0.662	0.653	0.651	0.706	0.608	3.4
	microRNA	Meta-learning	0.612	0.591	0.584	0.618	0.570	0.612	0.594	0.629	0.586	5.3
	Multi-omics	Direct learning	0.545	0.591	0.562	0.589	0.574	0.540	0.551	0.579	0.527	6.8
		Multitask learning	0.564	0.606	0.573	0.607	0.593	0.564	0.565	0.607	0.554	5.8
		Pretraining	0.653	0.678	0.642	0.688	0.662	0.652	0.658	**0.711**	0.591	2.5
		WO_similarity	0.649	**0.690**	0.642	0.677	0.657	**0.665**	0.655	0.707	0.627	2.6
		MMOSurv	**0.662**	**0.690**	**0.649**	**0.689**	**0.669**	**0.665**	**0.660**	0.710	**0.630**	**1.1**
20-shot	Gene	Meta-learning	0.637	0.679	0.634	0.685	0.664	0.658	0.651	0.709	0.608	3.4
	microRNA	meta-learning	0.613	0.593	0.587	0.620	0.573	0.612	0.598	0.628	0.587	5.7
	Multi-omics	Direct learning	0.555	0.626	0.578	0.620	0.594	0.551	0.565	0.599	0.528	6.7
		Multitask learning	0.579	0.636	0.588	0.634	0.609	0.573	0.579	0.627	0.559	5.6
		Pretraining	0.649	0.687	0.646	0.694	0.661	0.659	0.660	0.711	0.601	2.7
		WO_similarity	0.652	**0.695**	0.639	0.681	0.657	0.668	0.656	0.707	**0.629**	2.7
		MMOSurv	**0.665**	**0.695**	**0.654**	**0.695**	**0.670**	**0.673**	**0.661**	**0.713**	**0.629**	**1.0**

The average ranking of *C*-index for each method on the nine target cancer datasets is depicted in the rightmost column. Note that lower value is better. The best and second best results are highlighted in bold and underlined, respectively.

On the other hand, for multi-omics survival prediction, our MMOSurv outperforms the other methods. Direct learning that trains prediction model with very small training samples performs poorly on test dataset for all cancers, as one could expect. Compared with direct learning, the other multi-omics methods including MMOSurv, WO_similarity, regular pretraining, and multitask learning could obtain significant improvement on *C*-index and AUC values, which indicates that the knowledge from the relevant cancer multi-omics datasets can significantly enhance the performance of survival prediction model in multi-omics few-shot survival analysis problem. Moreover, our proposed MMOSurv method outperforms regular pretraining method and multitask learning method on most target cancer datasets. Specially, for BRCA, COAD, and ESCA datasets, MMOSurv method has a significant improvement over the regular pretraining method and multitask learning method. These results demonstrate that meta-learning is a remarkably more effective approach of knowledge transfer in few-shot problem of multi-omics survival analysis. Furthermore, MMOSurv explores the cross-correlations among different omics by the similarity constraint and achieves more satisfactory performance compared with WO_similarity method. To further compare different methods, we also reported the average ranking in terms of both *C*-index and AUC for each method over the nine datasets. From the average ranking perspective, it is obvious that our method MMOSurv is significantly superior to the other methods. To show the performance of MMOSurv with a large sample size, we added the experiments of MMOSurv with 100 training samples on the nine common target cancer datasets and reported the *C*-index values of different methods in Supplementary Table S2.

**Figure 3.**
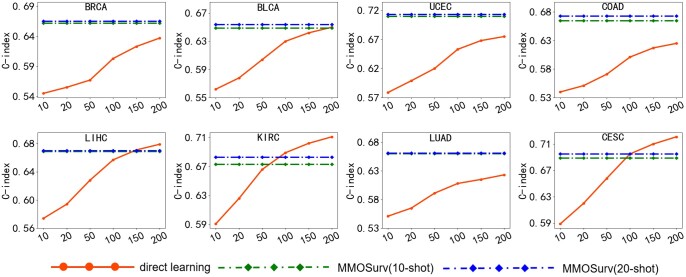
C-index for target cancer survival prediction, comparing direct learning with different sizes [10, 20, 50, 100, 150, 200] samples and MMOSurv with 10/20 samples.

To further illustrate the effectiveness of the MMOSurv method in multi-omics few-shot survival analysis problem, we compared MMOSurv based on only 10/20 training samples and direct learning based on large training samples. Specifically, we first drew six different sizes [10, 20, 50, 100, 150, 200] of training data from the common eight target cancer (except ESCA which only provides 164 samples in TCGA database) for direct learning, and then compared our MMOSurv method based on only 10/20 training samples from the target task with direct learning based on different sizes of training samples from the target task. As shown in Fig. 3, one could see that as the number of training samples gradually decreases, the performance of direct learning also drops. That is because the reduction of training samples leads to vast quantities of information loss, which makes the model more prone to overfitting and hardly learn well. Meanwhile, we can see that, for all cancer types, MMOSurv method with 10/20 training samples reaches competitive performances as direct learning with a significantly larger number of samples. Especially for BRCA, COAD, UCEC, and LUAD, MMOSurv can outperform direct training with 200 training samples by a large margin. For example, the mean *C*-index for BRCA is 0.637 for direct learning with 200 training samples and 0.662/0.665 for MMOSurv with 10/20 training samples; for COAD, the mean *C*-index is 0.625 for direct learning and 0.665/0.673 for MMOSurv; for UCEC, the mean *C*-index is 0.675 for direct learning and 0.710/0.713 for MMOSurv; for LUAD, the mean *C*-index is 0.623 for direct learning and 0.660/0.661 for MMOSurv. These results fully demonstrate that MMOSurv could solve the few-shot problem by effectively taking advantage of meta-knowledge across tasks from multi-omics data of relevant cancers.

### 3.4 Validation of MMOSurv on two rare cancers

**Figure 4.**
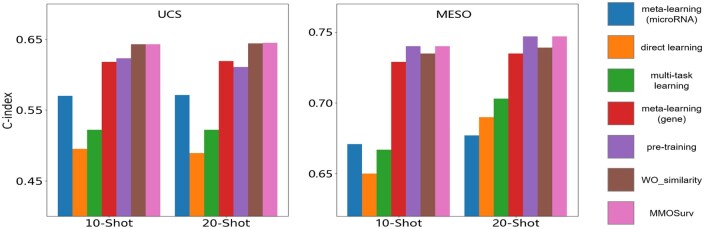
C-Index for survival prediction on the two rare cancer cohorts, comparing single-omics meta-learning, direct learning, multitask learning, regular pretraining, WO_similarity, and MMOSurv in few-shot (10/20) setting.

For evaluating our MMOSurv in real applications, we conducted validation on the two rare cancer cohorts: UCS and MESO. We compared our MMOSurv method with single-omics meta-learning, direct learning, multitask learning, regular pretraining, and WO_similarity methods in the 10-shot and 20-shot scenarios. Figure 4 and Supplementary Fig. S1 show the *C*-index and AUC values of different methods, respectively. From Fig. 4, we can find that multi-omics methods based on knowledge transfer including multitask learning, pretraining, WO_similarity and MMOSurv significantly outperform direct learning. For example, the mean *C*-index values for multitask learning on UCS and MESO datasets are 0.522 and 0.703 in the 20-shot scenario, while those for direct learning are 0.489 and 0.690. Meanwhile, the results show that multi-omics meta-learning method MMOSurv could achieve similar or better performance than regular pretraining and multitask learning. For UCS and MESO, the mean *C*-index values for MMOSurv are 0.645 and 0.747 in the 20-shot scenario, compared to 0.611 and 0.747 for regular pretraining. Taken together, the above analysis demonstrates that, compared to multitask learning and regular pretraining, meta-learning is a significantly promising approach for multi-omics few-shot survival prediction when leveraging high-dimensional multi-omics data from relevant cancers.

Moreover, for UCS and MESO, the mean *C*-index values for single-omics meta-learning (gene/microRNA) are 0.619/0.571 and 0.735/0.677 and the mean *C*-index values for WO_similarity are 0.644 and 0.739, in the 20-shot scenario. MMOSurv achieves significant improvement over single-omics meta-learning and WO_similarity methods, which indicates that meta-information of similarities and relationships between different omics data from relevant cancer datasets can help improve the performance on target cancer task with a few training samples. In addition to *C*-index, MMOSurv has satisfactory performance on AUC compared to other investigated methods, which reflects the superiority of the proposed method in the few-shot problem of multi-omics survival analysis.

### 3.5 Performance comparison with meta-learning on existing methods

MMOSurv is further assessed by comparing with some state-of-the-art methodologies, including survival convolutional neural networks (SurvCNN) (Kalakoti *et al.* 2021), deep survival machines (DSM) (Nagpal *et al.* 2021), and variable selection method (LAD_Network) (Ren *et al.* 2019). SurvCNN transforms multi-omics data into corresponding image representation and trains convolutional neural networks to predict the prognosis of cancer patients; DSM assumes the survival function of each individual as a weighted mixture of *K* fully parametric survival distributions to accommodate for heterogeneity among cancer patients; LAD_Network adopted a robust network‐constrained regularization to accommodate correlations among high-dimensional gene expressions for cancer prognosis. Although these methods have achieved satisfactory performance in survival analysis of large-scale datasets, they all perform poorly when there are a very few training samples. So, for fair comparison, we applied meta-learning strategy on the three methods, namely Meta_DSM, Meta_LAD_Network, and Meta_SurvCNN, for the few-shot setting. Table 3 reports the mean values of the *C*-index obtained by different methods with 20 training samples. From the experimental results listed in Table 3, we can see that MMOSurv explores the cross-correlations among different omics by the similarity constraint and achieves more satisfactory performance compared with single-omics meta-learning methods Meta_LAD_Network, Meta_DSM, and multi-omics meta-learning method Meta_SurvCNN.

**Table 3.** Performance comparison of existing methods with 20 training samples using *C*-index values.

Data	Method	BRCA	BLCA	LIHC	KIRC	COAD	UCEC	CESC	ESCA	LUAD	MESO	UCS
microRNA	Meta_LAD_Network	0.636	0.578	0.597	0.631	0.582	0.638	0.656	0.560	0.604	0.716	0.532
RNASeq	Meta_LAD_Network	0.587	0.601	**0.670**	0.655	0.575	0.605	0.647	0.539	0.559	0.739	0.553
microRNA	Meta_DSM	0.621	0.580	0.586	0.602	0.600	0.653	0.643	0.550	0.605	0.699	0.604
RNASeq	Meta_DSM	0.629	0.636	**0.670**	0.679	0.634	0.698	0.675	0.598	0.646	0.722	0.626
multi-omics	Meta_SurvCNN	0.609	0.612	0.665	0.651	0.590	0.653	0.627	0.592	0.622	0.698	0.594
multi-omics	MMOSurv	**0.665**	**0.654**	**0.670**	**0.683**	**0.673**	**0.713**	**0.695**	**0.629**	**0.661**	**0.747**	**0.645**

The best results are highlighted in bold.

### 3.6 External validation of MMOSurv

In addition, we performed external validation using the independent TARGET-AML dataset from Children’s Oncology Group biology studies and clinical trials (Bolouri *et al.* 2018), to further demonstrate the superiority of MMOSurv method in multi-omics few-shot problem. TARGET-AML dataset which includes genomic data (such as gene expression, microRNA expression, and DNA methylation) and clinical data of patients with acute myeloid leukemia, is publicly available in UCSC Xena database (https://xena.ucsc.edu/). The original data can be accessed at https://target-data.nci.nih.gov/Public/AML/. We take TCGA datasets as meta-training dataset and TARGET-AML dataset as meta-test dataset. We extract meta-knowledge across tasks drawn from TCGA datasets to make the model learn a suitable initialization parameter in the meta-learning phase, and fine-tune the model with a few training samples from TARGET-AML dataset in the final learning phase. We reported the *C*-index values of different methods (including single-omics meta-learning methods, direct learning, regular pretraining, multitask learning and MMOSurv) with 20 training samples in Supplementary Fig. S3. From the experimental results, we can see that MMOSurv achieves significant improvement over single-omics meta-learning methods, which suggests that exploring the meta-knowledge of cross-correlations among different omics from TCGA cancer datasets can help improve the performance on TARGET-AML cancer task with a few training samples. Moreover, it is of note that MMOSurv reaches the best *C*-index value of 0.632 among all multi-omics methods, outperforming direct learning, multitask learning and pretraining by approximately 6.9%, 6.8%, and 1.4%, respectively. The result demonstrates that meta-learning is a remarkably more effective approach of knowledge transfer in few-shot problem of multi-omics survival analysis.

## 4 Conclusions

Previous studies have shown that multi-omics methods could achieve a superior performance compared to single-omics methods for many tasks such as survival prediction. For large-scale datasets, multi-omics representation learning for survival analysis can recognize patient-distinguishable patterns and generate similar discriminative feature representations of multi-omics data for accurate survival prediction, but the approach does not work effectively on target task with small training samples, especially for rare cancers.

To address this issue, we proposed a novel multi-omics meta-learning method named MMOSurv for multi-omics few-shot survival analysis. MMOSurv learns the meta-knowledge extracted across tasks from multi-omics datasets of relevant cancers, to quickly recognize patient-distinguishable pattern for survival prediction on target cancer task with a few training samples. Specifically, MMOSurv first learns a suitable initialization of parameters for multi-omics survival model, from a variety of different tasks, and then adapts the parameters quickly and efficiently for the target cancer task with a very few training samples. Comprehensive experimental results on real-world datasets not only demonstrate that MMOSurv with a very few training data can obtain similar performance as learning from a significantly larger number of samples by leveraging high-dimensional multi-omics data from relevant cancers, but also show that MMOSurv achieves superior predictive performance over other state-of-the-art strategies such as multitask learning and pretraining in few-shot setting.

MMOSurv assesses the prognostic risk of cancer patients based on individual genomics data. In clinical practice, physicians can integrate the risk predictions from MMOSurv with standard clinicopathologic variables, such as tumor size and extent, to provide a more accurate personalized prognosis for cancer patients. Future research could explore the integration of multimodal data including genomic profiles, pathological images, clinicopathologic variables, and so on, to predict the survival risk of cancer patients more comprehensively and accurately with limited training data.

## Supplementary Material

btae684_Supplementary_Data

## Data Availability

The TCGA datasets are publicly available and can be obtained from: https://www.cancer.gov/ccg/research/genome-sequencing/tcga.
