# Supplementary material for: MMOSurv: meta-learning for few-shot survival analysis with multi-omics data
Source: Bioinformatics. 2024 Nov 19;41(1):btae684. doi: 10.1093/bioinformatics/btae684 (PMC11673192; doi:10.1093/bioinformatics/btae684)
Supplement: btae684_Supplementary_Data [file btae684_supplementary_data.pdf]

Supplementary Material

1 Evaluation of MMOSurv using AUC values

We evaluated the performance of MMOSurv approach in multi-omics few-shot survival prediction with another commonly used metrics: AUC. Table 1 and Figure 1 show AUC values of different methods on nine common cancer datasets and two rare cancer datasets in few-shot (10/20) setting, respectively. We can see that, compared with single-omics meta-learning methods, MMOSurv obtains higher AUC values, which indicates multi-omics meta-learning method can alleviate the omics-bias problem by exploring relationships and similarities among different omics data and achieve better performance. Moreover, our MMOSurv has satisfactory performance on AUC compared with the other multi-omics methods, which reflects the superiority of meta-learning in the few-shot problem of multi-omics survival analysis. Furthermore, MMOSurv explores the cross-correlations among different omics by the similarity constraint and has a significant improvement over WO_similarity on AUC. Taken together, the above analysis demonstrates that MMOSurv has significant advantages in the few-shot problem of multi-omics survival analysis.

Table 1: AUC of different approaches on nine common cancer datasets in few-shot (10/20) setting. The best and second best results are highlighted in bold and underlined respectively.

	Data	Method	BRCA	KIRC	BLCA	CESC	LIHC	COAD	LUAD	UCEC	ESCA	Avg.ranking
10-shot	gene	meta-learning	0.654	0.702	0.669	0.703	<u>0.714</u>	0.675	0.690	0.731	0.615	3.2
	microRNA	meta-learning	0.625	0.608	0.594	0.640	0.592	0.631	0.614	0.654	0.608	5.3
	multi-omics	direct learning	0.550	0.614	0.571	0.606	0.611	0.545	0.563	0.608	0.534	6.8
		multi-task learning	0.573	0.634	0.588	0.632	0.620	0.579	0.585	0.631	0.557	5.8
		pre-training	<u>0.665</u>	0.713	0.663	**0.714**	0.712	0.669	**0.701**	**0.736**	0.592	<u>2.7</u>
		WO_similarity	0.663	**0.726**	<u>0.678</u>	0.702	0.706	<u>0.692</u>	0.692	0.728	<u>0.636</u>	2.8
		MMOSurv	**0.676**	<u>0.724</u>	**0.691**	<u>0.713</u>	**0.718**	**0.694**	<u>0.698</u>	<u>0.733</u>	**0.640**	**1.4**
20-shot	gene	meta-learning	0.651	0.713	0.674	0.706	<u>0.717</u>	0.681	0.689	<u>0.735</u>	0.620	3.2
	microRNA	meta-learning	0.626	0.610	0.597	0.643	0.594	0.631	0.620	0.653	0.608	5.8
	multi-omics	direct learning	0.562	0.654	0.590	0.640	0.633	0.561	0.578	0.626	0.535	6.8
		multi-task learning	0.591	0.669	0.605	0.663	0.638	0.585	0.598	0.651	0.568	5.6
		pre-training	0.662	<u>0.725</u>	0.668	<u>0.719</u>	0.711	0.677	**0.701**	**0.736**	0.603	2.9
		WO_similarity	<u>0.667</u>	**0.733**	<u>0.675</u>	0.706	0.707	<u>0.693</u>	0.693	0.727	<u>0.639</u>	<u>2.6</u>
		MMOSurv	**0.682**	**0.733**	**0.696**	**0.720**	**0.721**	**0.699**	<u>0.698</u>	<u>0.735</u>	**0.641**	**1.2**

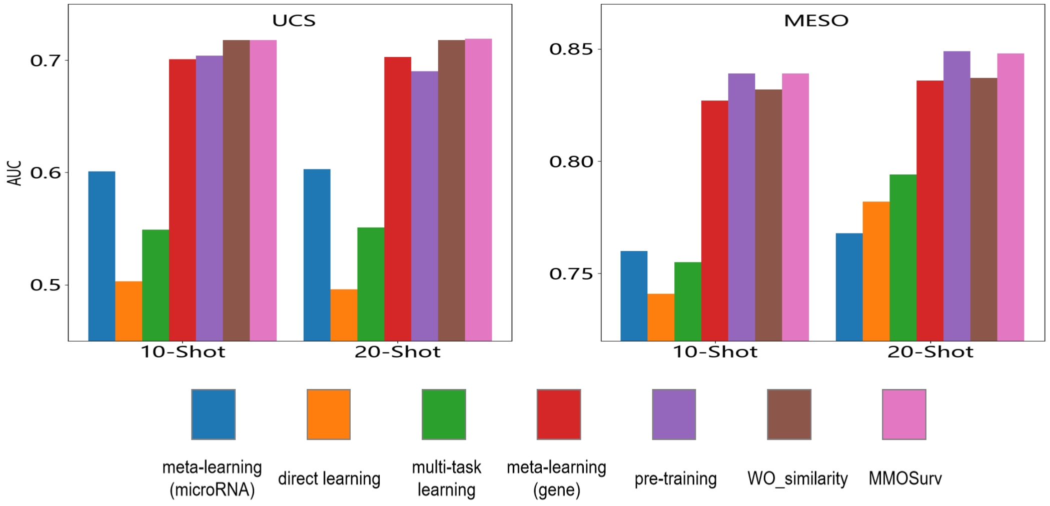

Figure 1: AUC for survival prediction on the two rare cancer cohorts, comparing single-omics meta-learning, direct learning, multi-task learning, regular pre-training, WO_similarity and MMOSurv in few-shot (10/20) setting.

2 Performance evaluation of MMOSurv with a large sample size

We added the experiments of MMOSurv with 100 training samples on the nine common target cancer datasets and showed the performance of MMOSurv with a large sample size. Table 2 shows the C-index values of different methods. Combining Table 2 of manuscript and supplementary Table 2, it is obvious that as the number of training samples gradually increases, the performance of all methods also improves. Meanwhile, we could see that MMOSurv and regular pre-training, compared with direct learning, could obtain better results in the scenario of 100 training samples, by taking advantage of the knowledge from the relevant cancer multi-omics datasets. More importantly, MMOSurv achieves more satisfactory performance, compared with alternative multi-omics methods including regular pre-training method and multi-task learning method. These results demonstrate that meta-learning is a remarkably effective approach of knowledge transfer in multi-omics survival analysis when leveraging high-dimensional multi-omics data from relevant cancers. Furthermore, MMOSurv has a significant improvement over single-omics meta-learning methods in the scenario of 100 training samples. This indicates that

MMOSurv can effectively take advantage of meta-information of similarities and relationships between different omics data from relevant cancer datasets, to improve prognosis prediction of the target cancer with a large sample size.

Table 2: Performance comparison of different approaches with 100 training samples using C-index values on the nine common target cancer datasets. The best and second best results are highlighted in bold and underlined respectively.

Data	Method	BRCA	KIRC	BLCA	CESC	UCEC	COAD	LUAD	LIHC	ESCA	Avg.ranking
gene	meta-learning	0.655	0.706	0.649	0.713	0.722	0.678	0.652	0.686	0.623	2.8
microRNA	meta-learning	0.623	0.620	0.604	0.638	0.649	0.616	0.599	0.607	0.626	4.5
multi-omics	direct learning	0.603	0.689	0.630	0.695	0.653	0.601	0.608	0.657	0.556	5.2
	multi-task learning	0.617	0.688	0.626	0.690	0.666	0.599	0.610	0.655	0.587	4.9
	pre-training	0.678	0.715	0.661	0.716	0.723	0.673	0.660	0.684	0.619	2.5
	MMOSurv	**0.685**	**0.717**	**0.666**	**0.723**	**0.725**	**0.687**	**0.663**	**0.688**	**0.647**	**1**

3 Significant test for the improvement of MMOSurv

We conducted t-tests to check whether there are statistically significant differences between the C-indices of the MMOSurv method and several alternative schemes, including single-omics meta-learning, WO_similarity, direct learning, regular pre-training and multi-task learning. T-test assumes that there is no difference in terms of C-index values between different methods and uses the t-distribution theory to infer the probability of differences occurring, thereby assessing the significance of differences between the two groups of data samples by p-value. The smaller the p-value, the more significant the difference between the two groups of data sample. We reported the p-values of significance tests of different scenarios (10/20) in Table 3. From the results we can see that, compared to other methods, MMOSurv can achieve statistically significant improvements in prediction performance by effectively taking advantage of meta-knowledge across tasks from multi-omics data of relevant cancers.

4 Comparison of Kaplan-Meier curves of different methods

To further evaluate the performance of MMOSurv in multi-omics few-shot survival analysis problem, we introduced Kaplan–Meier curves to test whether our method with 20 training

Table 3: Statistical significance tests of C-index of MMOSurv method and several alternative schemes in few-shot (10/20) scenario

	Data	gene	microRNA	multi-omics			
	Method	meta-learning	meta-learning	direct learning	multi-task learning	pre-training	WO_similarity
10-shot	BRCA	3.51e-64	7.31e-17	1.50e-255	1.64e-198	8.73e-04	1.13e-06
	BLCA	1.71e-165	4.04e-16	1.70e-213	2.12e-180	8.53e-04	2.01e-03
	KIRC	3.32e-244	3.33e-04	9.89e-149	1.51e-121	4.16e-01	8.46e-01
	COAD	1.14e-67	2.01e-05	3.87e-282	3.58e-207	8.73e-06	9.51e-01
	CESC	2.71e-149	3.62e-03	9.40e-173	9.43e-144	7.89e-01	1.56e-06
	ESCA	5.85e-85	2.36e-27	5.75e-309	1.47e-177	2.56e-70	1.22e-01
	LIHC	7.22e-237	1.37e-02	1.81e-162	9.18e-129	1.29e-02	3.25e-06
	LUAD	1.84e-144	3.56e-05	4.98e-279	9.28e-227	6.01e-01	5.97e-02
	UCEC	4.22e-136	1.65e-01	6.58e-241	9.03e-178	7.08e-01	3.73e-01
	UCS	1.63e-116	5.76e-18	0	1.12e-270	1.17e-11	3.40e-01
	MESO	2.44e-248	1.05e-11	1.08e-249	7.83e-180	7.13e-01	3.77e-03
20-shot	BRCA	4.73e-69	2.14e-24	6.04e-233	1.45e-163	7.15e-09	3.40e-07
	BLCA	9.34e-170	3.83e-19	4.04e-178	2.41e-147	4.75e-04	1.59e-11
	KIRC	6.95e-286	5.33e-08	8.18e-99	2.01e-79	5.34e-01	8.06e-02
	COAD	3.76e-93	5.99e-09	2.62e-276	2.61e-211	2.97e-07	8.03e-02
	CESC	8.22e-172	6.46e-06	2.70e-128	4.56e-101	7.11e-01	3.08e-10
	ESCA	7.98e-76	6.07e-24	7.61e-311	3.12e-164	3.46e-38	9.22e-01
	LIHC	4.07e-212	3.79e-02	4.66e-130	1.39e-91	1.53e-03	1.84e-06
	LUAD	3.56e-145	6.51e-06	3.36e-233	1.01e-184	6.40e-01	2.55e-02
	UCEC	1.16e-151	1.57e-01	6.57e-212	4.33e-138	4.51e-01	2.28e-02
	UCS	5.99e-126	2.49e-20	0	4.44e-281	1.81e-35	8.65e-01
	MESO	2.04e-266	3.48e-12	4.77e-160	3.04e-111	7.08e-01	8.68e-07

Table 4: Average and corresponding standard deviations of the C-index values for different methods on 1000 sampled tasks of target cancer in the few-shot (10/20) setting. The best and second best results are highlighted in bold and underlined respectively.

	Cancer	meta-learning (microRNA)	meta-learning (gene)	direct learning	multi-task learning	pre-training	WO_similarity	MMOSurv
10-shot	BRCA	0.612±0.005	0.639±0.004	0.545±0.005	0.564±0.005	0.653±0.004	0.649±0.004	**0.662±0.004**
	BLCA	0.584±0.003	0.632±0.002	0.562±0.004	0.573±0.004	0.642±0.002	0.642±0.002	**0.649±0.002**
	CESC	0.618±0.003	0.682±0.003	0.589±0.007	0.607±0.006	0.688±0.003	0.677±0.003	**0.689±0.003**
	COAD	0.612±0.005	0.653±0.004	0.540±0.005	0.564±0.005	0.652±0.004	**0.665±0.004**	**0.665±0.004**
	LIHC	0.570±0.004	0.662±0.003	0.574±0.007	0.593±0.006	0.662±0.003	0.657±0.003	**0.669±0.003**
	KIRC	0.584±0.003	0.664±0.003	0.591±0.006	0.604±0.005	0.671±0.002	**0.673±0.002**	**0.673±0.002**
	ESCA	0.586±0.002	0.608±0.002	0.527±0.003	0.554±0.004	0.591±0.002	0.627±0.002	**0.630±0.002**
	LUAD	0.594±0.003	0.651±0.002	0.551±0.004	0.565±0.004	0.658±0.002	0.655±0.002	**0.660±0.002**
	UCEC	0.629±0.005	0.706±0.004	0.579±0.008	0.607±0.007	**0.711±0.004**	0.707±0.004	0.710±0.004
	MESO	0.671±0.002	0.729±0.001	0.650±0.004	0.667±0.004	**0.740+0.001**	0.735±0.001	**0.740+0.001**
	UCS	0.570±0.005	0.618±0.004	0.495±0.005	0.522±0.005	0.623±0.005	**0.643±0.004**	**0.643±0.004**
20-shot	BRCA	0.613±0.005	0.637±0.004	0.555±0.005	0.579±0.005	0.649±0.004	0.652±0.004	**0.665±0.004**
	BLCA	0.587±0.003	0.634±0.002	0.578±0.004	0.588±0.003	0.646±0.002	0.639±0.002	**0.654±0.002**
	CESC	0.620±0.003	0.685±0.002	0.620±0.006	0.634±0.005	0.694±0.003	0.681±0.003	**0.695±0.003**
	COAD	0.612±0.005	0.658±0.004	0.551±0.005	0.573±0.005	0.659±0.004	0.668±0.003	**0.673±0.003**
	LIHC	0.573±0.004	0.664±0.003	0.594±0.005	0.609±0.005	0.661±0.003	0.657±0.003	**0.670±0.003**
	KIRC	0.588±0.003	0.671±0.003	0.626±0.004	0.635±0.004	0.682±0.002	0.679±0.002	**0.683±0.002**
	ESCA	0.587±0.003	0.608±0.002	0.528±0.003	0.559±0.003	0.601±0.002	**0.629±0.002**	**0.629±0.002**
	LUAD	0.598±0.003	0.651±0.002	0.565±0.004	0.579±0.004	0.660±0.002	0.656±0.002	**0.661±0.002**
	UCEC	0.628±0.005	0.709±0.004	0.599±0.006	0.627±0.006	0.711±0.004	0.707±0.004	**0.713±0.004**
	MESO	0.677±0.002	0.735±0.001	0.690±0.002	0.703±0.002	**0.747±0.001**	0.739±0.001	**0.747±0.001**
	UCS	0.571±0.004	0.619±0.004	0.489±0.004	0.522±0.005	0.611±0.004	0.644±0.004	**0.645±0.004**

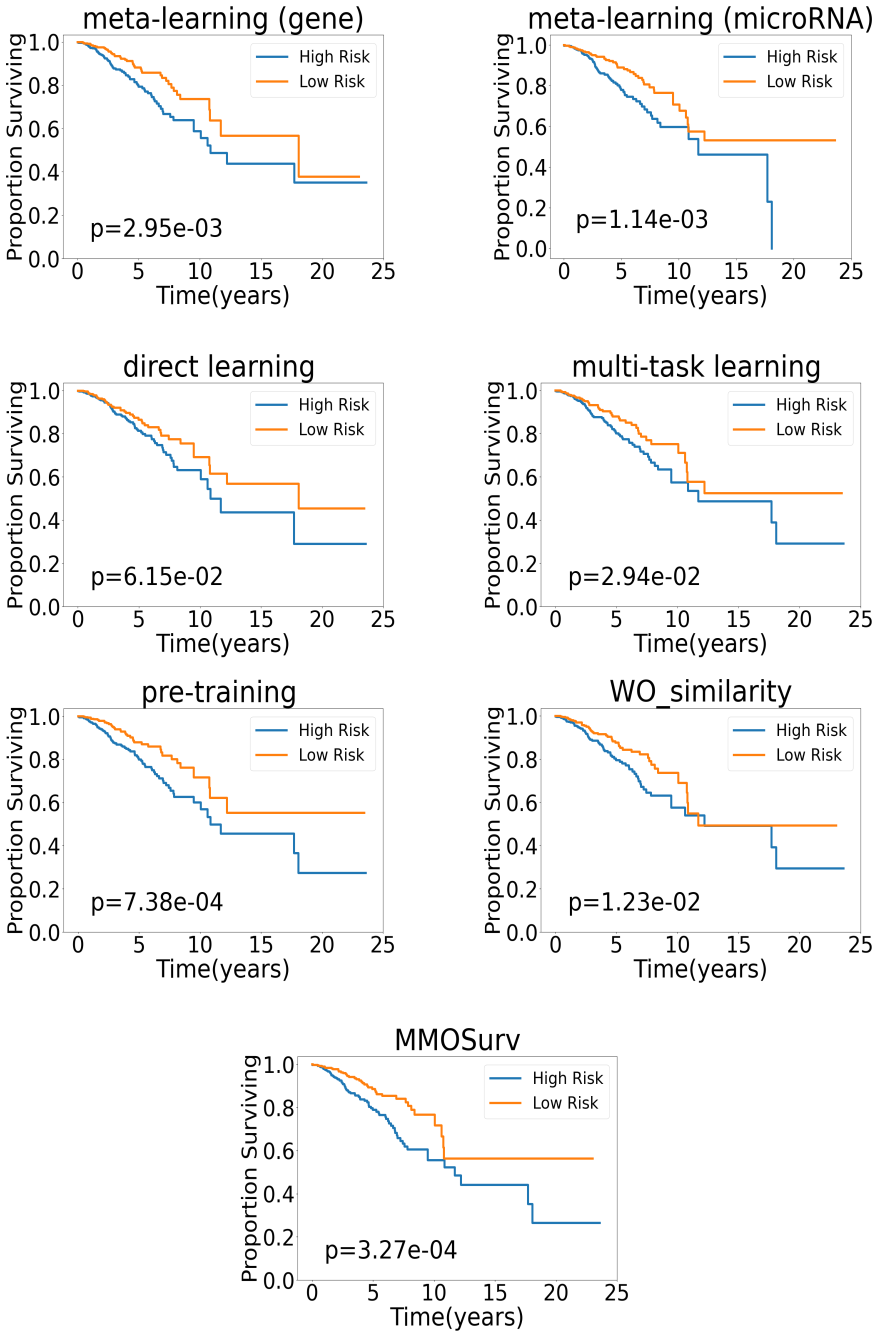

Figure 2: Kaplan–Meier curves of high- and low-risk groups predicted by different approaches with 20 training samples on BRCA dataset

samples can effectively distinguish high-risk group from low-risk group. First, we divided the test patients of breast cancer into low- and high-risk groups based on their predicted result. And then we plotted the corresponding K–M curves, and performed log-rank test to test whether there are remarkable differences between the survival curves of different risk groups. Figure 2 draw the K–M curves for the high- and low-risk groups predicted by different methods with 20 training samples of BRCA, and also showed the corresponding p-values of log-rank test. We can observe that MMOSurv obtained more significant log-rank p-value of 3.27e-04 than single-omics meta-learning (2.95e-03 and 1.14e-03). This implies that MMOSurv with multi-omics data enables better separation of low- and high-risk groups than only using single-omics data, in few-shot setting.

Furthermore, multi-omics learning methods based on knowledge transfer, including MMOSurv, WO_similarity, regular pre-training and multi-task learning, could obtain better results than direct learning by taking advantage of the knowledge from the relevant cancer multi-omics datasets. For example, multi-task learning obtained more significant log-rank p-values of 2.94e-02 than direct learning (6.15e-02). Moreover, among the multi-omics methods based on knowledge transfer, our multi-omics meta-learning MMOSurv produced the most significant p-value, and this shows that MMOSurv has better discrimination ability for high- and low-risk groups than WO_similarity, multi-task learning and pre-training, which reflects that MMOSurv is an effective method in few-shot problem of multi-omics survival analysis.

5 Long-term predictive analysis of MMOSurv

We categorized cancer patients of testing dataset into longer-term or shorter-term survivors by the criterion of 5-year survival, and assessed the prediction performance of MMOSurv on long-term survival data by estimating the C-index values between longer-term survivors and shorter-term survivors. Since longer-term survivors are rare in the ESCA, MESO, and UCS types of cancer, we only reported the results of different methods with 20 training samples for eight common cancers in Table 5. From the experimental results, we can see that multi-task learning, regular pre-training and MMOSurv, compared with direct learning, can better distinguish longer-term survivors from shorter-term survivors when leveraging high-dimensional multi-omics data from relevant cancers. Moreover, among the multi-omics methods based on

knowledge transfer, our proposed MMOSurv method outperforms regular pre-training method and multi-task learning method. Furthermore, MMOSurv achieves superior performance over single-omics meta-learning methods in the scenario of 20 training samples. Taken together, these results clearly demonstrate the superiority of MMOSurv in the few-shot problem of multi-omics survival analysis.

Table 5: Performance comparison of different approaches with 20 training samples using the C-index values between longer-term survivors and shorter-term survivors. The best and second best results are highlighted in bold and underlined respectively.

Data	Method	BRCA	KIRC	BLCA	CESC	COAD	UCEC	LUAD	LIHC
gene	meta-learning	0.641	<u>0.716</u>	0.634	<u>0.701</u>	<u>0.697</u>	0.777	0.699	<u>0.735</u>
microRNA	meta-learning	0.606	0.609	0.596	0.634	0.619	0.680	0.651	0.619
multi-omics	direct learning	0.555	0.657	0.605	0.644	0.578	0.653	0.580	0.623
	multi-task learning	0.585	0.671	0.588	0.659	0.617	0.681	0.607	0.650
	pre-training	<u>0.648</u>	**0.729**	<u>0.681</u>	<u>0.701</u>	0.684	<u>0.778</u>	**0.712**	0.732
	MMOSurv	**0.665**	**0.729**	**0.691**	**0.713**	**0.701**	**0.780**	<u>0.707</u>	**0.741**

6 Univariate and multivariate cox proportional hazards analysis

To evaluate the independent prognostic power of risk predicted by MMOSurv with 20 training samples, we performed univariate and multivariate Cox proportional hazards analysis of the predicted risk and other standard clinicopathologic variables in breast cancer prognosis, including age at diagnosis, histologic grade, extent of tumor (T stage), lymph node involvement (N stage) and presence of metastasis (M stage). Table 6 reported the results of univariate and multivariate analysis based on different factors. From the results, we can see that only the p-value of predicted risk factor is less than 0.005 in both univariate and multivariate analysis, which indicates that the predicted risk factor could retain strong and significant independent prognostic factor when correcting for other clinicopathologic variables. Taken together, the above analysis demonstrates that our MMOSurv method has great predictive power for survival in few-shot setting.

Table 6: Hazard ratios for univariate and multivariate cox proportional hazards analysis on BRCA dataset

		Univariate			Multivariate		
Variable		Hazard ratio	95%CI	P-value	Hazard ratio	95%CI	P-value
Age	$\leq 50/>50$	1.48	0.99-2.23	0.06	1.85	1.22-2.81	<0.005
Grade	$\leq II/>II$	2.25	1.55-3.28	<0.005	2.31	1.02-5.24	0.04
T Stage	$\leq T2/>T2$	1.58	1.03-2.42	0.04	0.91	0.47-1.76	0.78
N Stage	$\leq N1/>N1$	2.01	1.29-3.11	<0.005	1.04	0.47-1.29	0.92
M Stage	M0/MX	0.70	0.32-1.51	0.36	0.69	0.32-1.49	0.34
MMOSurv	Low risk/High risk	2.40	1.62-3.56	<0.005	2.48	1.66-3.70	<0.005

7 Complexity comparison

To assess the computational cost of MMOSurv method, we compared the time and space complexity of two multi-omics meta-methods, MMOSurv and Meta_SurvCNN.We use the number of learned parameters of MMOSurv and Meta_SurvCNN to characterize the space complexity of different methods. MMOSurv has 6.74MB parameters to learn, which is approximately 5.5 times of the number of parameters of Meta_SurvCNN. To assess the time complexity of MMOSurv and Meta_SurvCNN, we calculated the time it takes for each method to predict the risk of cancer patients during the testing phase. MMOSurv needs 19ms during testing on a system running Linux with CPU Intel(R) Core(TM) i9-12900K and GPU NVIDIA GeForce RTX 3090, compared with 178ms of Meta_SurvCNN. Meta_SurvCNN transforms multi-omics data into corresponding image representation and adopts parameter-sharing CNN networks to reduce the space complexity of the model. Although MMOSurv has more parameters to learn, MMOSurv can run faster and obtain more favorable performance.

8 Sensitivity analysis for the dimension of the embedding representations

In the revised manuscript (section 2.1), we set the dimension of the low-dimensional embedding representations $Z^{(i)}(i \in \{1, 2\})$ for gene expression data and microRNA expression data to 80.

We added sensitivity analysis for the dimension of the low-dimensional embedding representations, and checked the performance of MMOSurv in few-shot setting when the dimension of the embedding representations is 60, 80, and 100, respectively. Table 7 reported the results of the MMOSurv method with 20 training samples on different dimensions of the embedding representations. From the experimental results, we can see that although the performance of MMOSurv fluctuates with the change of the dimension of the embedding representations in few-shot scenario, it tends to be robust overall.

Table 7: Performance comparison of MMOSurv with 20 training samples on different dimensions of the embedding representation using C-index values

Dim	BRCA	KIRC	BLCA	CESC	LIHC	COAD	LUAD	UCEC	ESCA	MESO	UCS
60	0.662	0.694	0.653	0.696	0.662	0.673	0.656	0.709	0.621	0.741	0.652
80	0.665	0.695	0.654	0.695	0.670	0.673	0.661	0.713	0.629	0.740	0.645
100	0.666	0.698	0.654	0.693	0.666	0.674	0.663	0.714	0.636	0.746	0.652

9 External validation of MMOSurv

We performed external validation using the independent TARGET-AML dataset from Children's Oncology Group (COG) biology studies and clinical trials [1], to further demonstrate the superiority of MMOSurv method in multi-omics few-shot problem. TARGET-AML dataset which includes genomic data (such as gene expression, microRNA expression, and DNA methylation) and clinical data of patients with acute myeloid leukemia, is publicly available in UCSC Xena database (https://xena.ucsc.edu/). The original data can be accessed at https://target-data.nci.nih.gov/Public/AML/. We take TCGA datasets as meta-training dataset and TARGET-AML dataset as meta-test dataset. In the meta-learning method, the meta-learning stage extracts meta-knowledge across tasks drawn from TCGA data to make the model learn a suitable initialization parameter. The final training and testing are conducted on the independent dataset. The following Figure 3 shows the C-index values of different methods (including single-omics meta-learning methods, direct learning, regular pre-training, multi-task learning and MMOSurv) in the scenarios with 20 training samples. From the experimental results, we can see that MMOSurv achieves significant improvement over single-omics meta-learning meth-

ods, which suggests that exploring the meta-knowledge of cross-correlations among different omics from TCGA cancer datasets can help improve the performance on TARGET-AML cancer task with a few training samples. Moreover, it is of note that MMOSurv reaches a superior C-index of 0.632 among all multi-omics methods, outperforming direct learning, multi-task learning and pre-training by approximately 6.9%, 6.8% and 1.4%, respectively. The result demonstrates that meta-learning is a remarkably more effective approach of knowledge transfer in few-shot problem of multi-omics survival analysis.

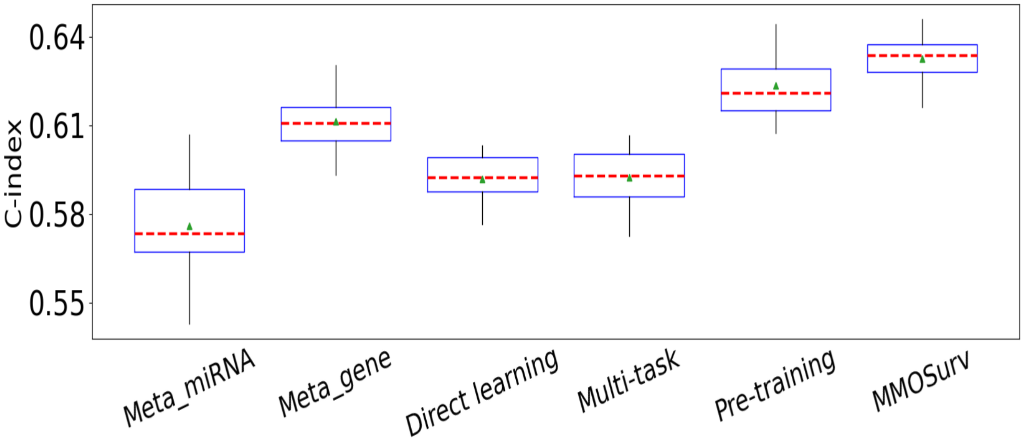

Figure 3: C-index for survival prediction on the independent TARGET-AML dataset, comparing small (20) size sample single-omics meta-learning (Meta_gene and Meta_miRNA), direct learning, multi-task learning, regular pre-training and MMOSurv.

10 Comparison of different imputation strategies on the performance of MMOSurv

In addition to the median imputation strategy mentioned in the manuscript, we also explored mean and k-nearest neighbors strategies to impute missing data, to assess the impact of different imputation strategies on model performance. The following Table 8 lists the results of these different imputation strategies. The experimental results show that different imputation

strategies produce similar results for MMOSurv, implying its robustness regarding missing value imputation strategies.

Table 8: Comparison of different imputation strategies on the performance of MMOSurv with 20 training samples using C-index values

Strategy	BRCA	KIRC	BLCA	CESC	LIHC	COAD	LUAD	UCEC	ESCA	MESO	UCS
KNN(K=2)	0.659	0.690	0.653	0.696	0.674	0.666	0.660	0.700	0.636	0.638	0.640
Mean	0.656	0.690	0.645	0.700	0.675	0.668	0.663	0.705	0.630	0.743	0.642
Median	0.665	0.695	0.654	0.695	0.670	0.673	0.661	0.713	0.629	0.740	0.645

11 MMOSurv on different types of omics data

MMOSurv is further assessed on multi-omics few-shot data with DNA methylation, specifically, including gene expression, microRNA expression and DNA methylation. Similar to prior works, for DNA methylation data, we filtered out the low information burden variables whose values remain almost unchanged across samples to reduce the number of noise-sensitive features, by which the top 10,000 features are chosen for further study. We performed MMOSurv method in single-omics, pairwise two-omics, and three-omics settings. We reported the results in the following Table 9 for the three settings, and marked the multi-omics results in bold when they have improvement over each corresponding single-omics data. We can see that compared with single-omics meta-learning methods, MMOSurv with two-omics and three-omics could both exhibit better performance in most cases. This indicates that MMOSurv can effectively take advantage of meta-information of similarities and relationships between different omics data from relevant cancer datasets.

From the experimental results listed in Table 9, it is obvious that MMOSurv with any two-omics shows remarkable improvement over single-omics meta-learning methods. Meanwhile, when there are lack of consistency discrimination information between omics, such as gene expression and DNA methylation, the performance of the MMOSurv method has decreased. Furthermore, from these few-shot survival experiment on two and three omics, we see that, MMOSurv with three omics has marginal improvement on two omics, for cancer types KIRC, COAD, ESCA, UCS and MESO. This is not surprising, because the more complex the network

structure is, the more likely it is that overfitting will occur in final learning with the limited training samples.

Table 9: C-indices by MMOSurv method on single-omics, two-omics and three-omics data with 20 training samples

Data	RNASeq	microRNA	DNA Methylation	RNASeq and microRNA	RNASeq and DNA Methylation	DNA Methylation and microRNA	three omics
BRCA	0.644	0.590	0.632	**0.652**	**0.655**	**0.638**	**0.655**
BLCA	0.640	0.572	0.559	**0.657**	0.632	**0.586**	**0.648**
KIRC	0.704	0.587	0.580	**0.714**	**0.713**	**0.603**	**0.719**
COAD	0.654	0.620	0.582	**0.673**	**0.668**	**0.627**	**0.678**
UCEC	0.689	0.595	0.618	**0.695**	**0.698**	**0.621**	**0.695**
CESC	0.690	0.615	0.639	**0.700**	**0.690**	0.634	**0.698**
ESCA	0.603	0.591	0.589	**0.620**	**0.616**	**0.613**	**0.628**
LUAD	0.634	0.586	0.570	**0.644**	0.625	**0.593**	**0.634**
LIHC	0.662	0.570	0.583	**0.669**	0.653	**0.595**	**0.666**
MESO	0.732	0.675	0.667	**0.743**	**0.741**	**0.685**	**0.749**
UCS	0.612	0.586	0.643	**0.634**	**0.662**	**0.668**	**0.670**

12 Evaluation metrics

In this study, we evaluated the performance of MMOSurv approach in multi-omics few-shot survival prediction with two metrics: C-index and AUC [2]. The C-index is the most widely used metric for evaluating prognostic models in survival analysis and is calculated as Equation (1). It assesses the predictive ability of the survival model by estimating the probability of concordance between the ordering of predicted results and ground truth results. The C-index value falls in the range $[0, 1]$, and a C-index value of 1.0 indicates that the predicted model always assigns lower risk to the patient of longer survival time.

$$\text{C-index} = Prob\{\beta^T z_i^h > \beta^T z_j^h | O_j > O_i, \delta_i = 1\}, \tag{1}$$

Another evaluation metric, Area Under Curve (AUC), quantifies the ranking quality at the event-time level. It is formulated as:

$$\text{AUC} = \frac{1}{num} \sum_{t \in Y} \sum_{i:O_i<t} \sum_{j:O_j>t} I(\beta^T z_i^h > \beta^T z_j^h) \tag{2}$$

where Y and num represent the set of all possible event times in the dataset, and the cumulative number of comparable pairs, respectively, and $I(\cdot)$ is the indicator function. Similarly, the value of AUC also ranges from 0 to 1 and higher value is better.

References

[1] Hamid Bolouri, Jason E Farrar, Timothy Triche Jr, Rhonda E Ries, Emilia L Lim, Todd A Alonzo, Yussanne Ma, Richard Moore, Andrew J Mungall, Marco A Marra, et al. The molecular landscape of pediatric acute myeloid leukemia reveals recurrent structural alterations and age-specific mutational interactions. Nature medicine, 24(1):103–112, 2018.

[2] Hung-Chia Chen, Ralph L Kodell, Kuang Fu Cheng, and James J Chen. Assessment of performance of survival prediction models for cancer prognosis. BMC medical research methodology, 12(1):1–11, 2012.
